# Perforation of the Colon During Imatinib Mesylate (Gleevec) Treatment in a Patient with Chronic Myeloid Leukemia (CML)

**DOI:** 10.7759/cureus.660

**Published:** 2016-06-27

**Authors:** Najla El Jurdi, Mark Bankoff, Andreas Klein, Muhammad W Saif

**Affiliations:** 1 Medicine, Tufts Medical Center; 2 Hematology/Oncology, Tufts Medical Center

**Keywords:** gastrointestinal stromal tumors, imatinib, tyrosine kinase inhibitor, perforation, anti vegf

## Abstract

Imatinib (Gleevec; STI-571) is a tyrosine-kinase inhibitor (TKI) used in the treatment of multiple cancers, most notably Philadelphia chromosome-positive (Ph+) chronic myelogenous leukemia (CML) as well as gastrointestinal stromal tumor (GIST). The most common adverse effects with imatinib include superficial edema, muscle cramps, musculoskeletal pain, rash, fatigue, headache, and gastrointestinal side effects. Less frequent side effects include pancytopenia, febrile neutropenia, flushing, and liver function test abnormalities. Very rare side effects include secondary malignancies, Sweet’s syndrome, angioedema, or cardiac arrest. We report the first case report of gastrointestinal perforation complicating imatinib treatment for CML. Unlike other antiangiogenic TKIs such as sunitinib or sorafenib that target vascular endothelial growth factor (VEGF) and known to cause gastrointestinal perforation, imatininib is a TKI with no known anti-VEGF activity, and so it remains unclear how imatinib would be associated with developing this life threatening complication. However, physicians caring for patients of imatinib should be aware of this potential toxicity. We suggest that careful attention and an appropriate clinical evaluation are required for patients presenting with gastrointestinal symptoms during imatinib treatment.

## Introduction

In May 2001, imatinib mesylate was approved by the FDA for the treatment of patients with chronic myeloid leukemia (CML) and became the first real triumph of the targeted cancer therapy era [1]. Imatinib mesylate is a 2-phenylaminopyrimidine compound that specifically interacts with the adenosine triphosphate (ATP) binding site of multiple tyrosine kinases including BCR-ABL (an aberrant tyrosine kinase resulting from a fusion protein product of the acquired Philadelphia chromosome identified in greater than 90% of patients with CML), ABL-related gene product (ARG), and certain subgroup III receptor tyrosine kinases (c-kit receptor, platelet-derived growth factor PDGF receptor, and stem cell factor receptor) [1-2]. Following its approval for treatment of patients with CML in blast crisis, accelerated phase or chronic phase after failure of IFN-a therapy, imatinib was also approved for the treatment of unresectable/metastatic malignant gastrointestinal stromal tumors (GIST) that express the tyrosine kinase receptor c-kit [3]. After more than a decade now of its introduction into the clinical practice, there is a better understanding of both the short- and long-term consequences and adverse effects of imatinib. Some of the most frequently reported adverse effects seen in patients treated with imatinib include superficial edema, muscle cramps, musculoskeletal pain, rash, fatigue, headache, gastrointestinal side effects including abdominal pain and joint pain in 10% of the cases. Less frequent side effects with reported incidence of 1-10% include pancytopenia, febrile neutropenia, flushing, and liver function test abnormalities [2-3].

We report a case of a patient who developed colorectal perforation during therapy with imatinib, which is a very rare side effect of other anti-angiogenic agents but not to imatinib and imatinib-like agents.

## Case presentation

A 60-year-old Caucasian male with a past medical history of hypertension and coronary artery disease initially presented with acute onset left arm weakness and left facial droop. He was first evaluated by a local health care facility where a CT scan of his head was done and revealed a right frontal intraparenchymal hemorrhage with surrounding edema, mass effect, and midline shift. He was transferred to the neurosurgery service at our medical center for further management. The patient had been seen at an outside institution a week prior to admission for recent back pain and was found to have an elevated international normalized ratio (INR) of 6.1 at that time. He denied fevers, chills, shortness of breath, chest pain, early satiety, nausea, vomiting, or abdominal discomfort at admission. He had a history of hypertension and coronary artery disease with ischemic cardiomyopathy and left ventricular ejection fraction of 25% with a left ventricular aneurysm and apical thrombus for which he has been on chronic anticoagulation. Medications on admission included aspirin, lisinopril, allopurinol, metoprolol, niacin, simvastatin, warfarin, venlafaxine, and Ambien as needed. He had no known allergies. He lived with his wife, used to drink one or two beers a couple of times a week, and denied any recreational drugs use. One of his sons was diagnosed with acute lymphoblastic leukemia (ALL) at the age of 13 and was 21 years out from his treatment. He has no family history of hematological or other neoplastic diseases. The patient agreed to participate and was explained the nature and objectives of this study, and informed consent was formally obtained. No reference to the patient's identity was made at any stage during data analysis or in the report.

His physical examination did not reveal any lymphadenopathy or splenomegaly. No focal neurological deficit was noticed. Initially, the patient’s intracranial hemorrhage was thought to be in the setting of a supratherapeutic INR due to chronic anticoagulation with coumadin for left ventricular apical thrombus. However, his admission laboratory data showed leukocytosis with a white blood cell count (WBC) of 298,000/cmm with early forms (promyelocytes 7%, myelocytes 8%, metamyelocytes 4%, blasts 1%). The differential count at that time was neutrophil 61%, band 11%, lymphocytes 2%, basophils 2%, eosinophils 2% with platelet count of 76,000/cmm, hemoglobin of 10.1 gm/dL. Due to the concern of probable leukostasis secondary to leukocytosis contributing to the intracranial hemorrhagic stroke, the patient underwent leukapheresis (two units of whole blood phlebotomized) and received two units of red cell transfusion which resulted in a significant decline in WBC count to 228,000/cmm. Flow cytometry and FISH revealed 9:22 translocation; Philadelphia chromosome was consistent with the diagnosis of CML. A bone marrow biopsy also confirmed the diagnosis of CML, and there was no evidence of increased blasts in the bone marrow, indicating that he was in the chronic phase of CML. Quantitative PCR showed 43.1% B2A2/GUS. During hospitalization, aspirin and warfarin were stopped, and he was managed conservatively with platelet transfusions and close observation. He did not require surgical intervention and began rehabilitation therapy for his left hemiparesis. He was started on hydroxyurea (Hydrea) two grams daily titrated up to two grams twice daily followed by imatinib (Gleevec) 400 mg orally daily.

The patient returned for a follow-up to the hematology clinic two weeks after discharge; labs showed a WBC of 3,500/cmm with no immature forms, and the differential count was neutrophil 60%, band 0%, lymphocytes 29%, basophils 1%, eosinophils 1% with platelets of 50,000/cmm, hemoglobin of 12.1 g/dL. Peripheral smear showed decreased platelet count with normal-looking neutrophils without any immature forms such as myeloblasts or promyelocytes and no evidence of dysplasia such as hypogranulated neutrophils, basophilic stippling in the red cells, or giant platelets. He was in hematologic remission, and the dose of Gleevec was decreased to 300mg daily from 400mg daily in the setting of thrombocytopenia that was initially attributed to Gleevec.

The patient returned to the clinic after two months for follow-up; Gleevec was increased back to 400mg daily since there was no significant change in his platelet count by decreasing the dose, and his platelet count at that time was 65,000/cmm. He was then followed up in clinic every two months, and quantitative FISH demonstrated a complete cytogenetic response at nine months. The patient had a decrease in BCR-ABL PCR transcript on testing at twelve months follow-up from 43.1% to 0.29%, demonstrating a major molecular response.

The patient returned to the clinic for an interval follow-up. Following several months of conservative management and evaluation of worsening lower back pain over five months that was initially attributed to long hours of driving, he had a CT scan done at an outside hospital which demonstrated a likely perforation of the rectosigmoid colon, and was hospitalized for management. Upon admission, Gleevec was discontinued with the identification of bowel perforation as a potential rare but serious complication of Gleevec. He continued to have episodes of pain up to 8-10/10 in intensity and hence transferred to our facility. A repeat CT scan of the abdomen and pelvis with contrast showed evidence for extravasation of the administered rectal contrast into the perirectal region. Multiple pockets of gas were seen posterior and lateral to the distal rectum in the presacral region and in the region of the rectal wall with noted surrounding inflammatory changes but no organized fluid collection. This was followed by a colonoscopy with special care taken to avoid overinflation or advancement of the colonoscope beyond the affected region based on CT findings. However, colonoscopy did not reveal perforation. He was managed conservatively after consulting surgery and finally discharged home in improved condition. Also, he was resumed on Gleevec.

One month later, he presented with recurrent lower GI bleeding. A repeat colonoscopy was done that reportedly revealed a rectosigmoid fistula into the retroperitoneal space, confirming the previous perforation. Gleevec was discontinued indefinitely at that time.

He had a follow-up in 14 days with a flexible sigmoidoscopy which showed an edematous mucosa 10 cm from the entry point, and biopsy was consistent with chronic nonspecific inflammation and mild focal hyperplastic changes and reportedly negative for malignancy.

Unfortunately, he again presented to the emergency department with similar symptoms of rectal pain and bleeding. CT scan at that time showed the possibility of abscess in the same area. An examination under anesthetic (EUA) along with incision and drainage of a perirectal abscess was performed along with a drain placement. He was followed up in four weeks both in hematology and surgery clinics. At this time, a follow-up CT scan of the pelvis showed that the cavity is smaller, but the track is still open which was thought to be reasonable since the drain fell out three days prior to presentation. The patient had no new accumulation of pus, no fevers, no chills, no back pain, and the perianal irritation was improving. 

## Discussion

This case is the first report of gastrointestinal perforation complicating imatinib treatment for CML. Imatinib is generally thought to be a well-tolerated drug, but most patients experience at least one Grade 1-2 adverse event during their treatment course. Gastrointestinal side effects of imatinib include mostly nausea, diarrhea, abdominal pain, dyspepsia, flatulence, abdominal distention, constipation, stomatitis/mucositis, taste disturbances, and transaminitis with fewer incidences of elevations in alkaline phosphatase and bilirubin. Patients with CML  have different clinical findings that vary depending on the stage of disease at diagnosis. In reports, up to 50% of patients are asymptomatic and incidentally diagnosed on routine blood tests [4]. Symptomatic patients mostly have systemic symptoms such as fatigue, malaise, weight loss, excessive sweating, and abdominal fullness, in addition to bleeding episodes due to platelet dysfunction. Gastrointestinal symptoms in CML patients include abdominal pain and discomfort with early satiety, due to the enlarged spleen with or without perisplenic inflammation/infarction. Gastrointestinal perforation with imatinib has been rarely reported in the literature usually in association with advanced GIST [5], although in the literature, the most common cases of bowel perforation occur in association with bevacizumab, sunitinib, and sorafenib used for the treatment of colorectal cancer, renal cell carcinoma and other solid malignancies [6-8]. All vascular endothelial growth factor (VEGF) inhibitors including bevacizumab (a monoclonal antibody approved for the treatment of non-small cell lung cancer, renal cell carcinoma, glioblastoma multiforme, ovarian, and metastatic colorectal cancer), aflibercept (recombinant fusion protein which comprises of portions of binding domains for VEGF receptors 1 and 2 attached to the Fc portion of human IgG1 that is approved with combination chemotherapy for treatment of recurrent metastatic colorectal cancer), and antiangiogenic TKI can potentially cause gastrointestinal perforation and fistula formation. Bevacizumab, used to treat metastatic colorectal cancer and epithelial ovarian cancers patients, has been most commonly associated with gastrointestinal perforation in the absence of predisposing risk factors. The literature reports four cases of gastrointestinal perforation in patients receiving thalidomide for the treatment of multiple myeloma [9].

The mechanism(s) by which these drugs cause or contribute to gastrointestinal perforation is not completely understood. It has been proposed that VEGF-targeted therapies lead to perforation through multiple mechanisms [10]:

(1) association between bevacizumab with ulcer development--especially in the areas of pre-existent mucosal lesions--might eventually lead to gastrointestinal perforation [9],

(2) it is also postulated that drugs such as bevacizumab result in cholesterol emboli syndrome, which features hypertension and eosinophilia that may subsequently lead to mesenteric ischemia and perforation [9],

(3) direct and indirect inhibition of angiogenesis with VEGF inhibitors result in reduction of normal blood vessels and vascular density as well as impaired healing of bowel injury, which might contribute to this complication [9], and

(4) gastrointestinal perforation might occur secondary to necrosis of ovarian cells that have invaded the bowel serosa.

Unlike other antiangiogenic TKIs such as sunitinib or sorafenib that target VEGF and therefore could be thought to cause gastrointestinal perforation through the same postulated mechanisms as above, imatininib is a TKI with no known anti-VEGR activity, and so it remains unclear how imatinib would be associated with developing this life-threatening complication [10]. 

## Conclusions

In conclusion, imatinib should be considered as a cause of unexpected colon perforation and subsequent peritonitis. Hence, careful attention and appropriate clinical evaluation are required for patients presenting with gastrointestinal symptoms during imatinib treatment. However, physicians caring for patients of imatinib should be aware of this potential toxicity.
